# The knowledge of memory aging questionnaire (KMAQ) in a Brazilian sample: a questionnaire for informants to recognize early signs of dementia

**DOI:** 10.1590/1980-5764-DN-2022-0090

**Published:** 2023-05-29

**Authors:** Mariel Carolina Montiel-Aponte, Paulo Henrique Ferreira Bertolucci, Gustavo Gil Velho Rocha

**Affiliations:** 1 Universidade Federal de São Paulo, Escola Paulista de Medicina, Programa de Pós-Graduação em Neurologia e Neurociências, São Paulo SP, Brazil.; 2 Universidade Federal de São Paulo, Escola Paulista de Medicina, Departamento de Neurologia, São Paulo SP, Brazil.; 3 Universidade Federal de São Paulo, Escola Paulista de Medicina, Hospital São Paulo, Ambulatório de Neurologia Comportamental, São Paulo SP, Brazil.; 4 Universidade Federal de São Paulo, Escola Paulista de Medicina, São Paulo SP, Brazil.

**Keywords:** Knowledge of Memory Aging Questionnaire, Surveys and Questionnaires, Caregivers, Dementia, Questionário de Conhecimento de Memória e Envelhecimento, Inquéritos e Questionários, Cuidadores, Demência

## Abstract

Till present, only a few countries have developed support programs for caregivers and families of patients with dementia aimed to improve knowledge, skills, and strategies to deal with the patient's symptoms. However, prior to offering this special support, it is important to identify beliefs and thoughts shared by informants related to cognition in elderly people. Questionnaires are instruments that allow having this information, such as the Knowledge of Memory Aging Questionnaire (KMAQ), which was designed to assess normal and pathological changes in the aging process.

**Objective:**

The aim of this study was to assess the knowledge about cognition, aging, and dementia as evaluated by the KMAQ in people who are in contact with elderly people, with and without cognitive impairment.

**Methods:**

A total of 78 relatives and caregivers of elderly patients were classified into two groups: group 1: relatives of patients with dementia (n1=48), and group 2: relatives of patients without cognitive impairment (n2=30). They were asked to answer some questionnaires about dementia, including the KMAQ.

**Results:**

Comparing the questionnaire's scores for normal cognitive changes items (g1: 0.53 *vs.* g2: 0.53, p-value: 0.99) did not show differences between the knowledge in both groups, nor shows the scores for pathological cognitive changes items (g1: 0.55 *vs.* g2: 0.55, p-value: 0.969).

**Conclusions:**

It seems that being in contact with dementia does not improve knowledge about it. Knowledge of normal changes in cognition could make it possible to recognize “red flags” suggestive of neurodegenerative processes, allowing for earlier diagnosis and more options for treatment.

## RESUMO.

Até o presente momento, poucos países desenvolveram programas destinados a cuidadores e familiares de pacientes com demência, objetivando o aprimoramento de conhecimentos, habilidades e estratégias para lidar com os sintomas dos pacientes. Entretanto, antes de prestar esse suporte especial, faz-se importante identificar crenças e pensamentos compartilhados por informantes acerca da cognição em pessoas idosas. Os questionários são instrumentos que permitem obter essa informação, a exemplo do Questionário de Conhecimento de Memória e Envelhecimento (KMAQ, *sigla em inglês*), que foi elaborado para avaliar mudanças normais e patológicas no processo de envelhecimento.

**Objetivo:**

O objetivo deste estudo foi avaliar o conhecimento sobre cognição, envelhecimento e demência pela aplicação do KMAQ em pessoas que estão em contato com pessoas idosas com e sem comprometimento cognitivo.

**Métodos:**

Setenta e oito familiares e cuidadores de pacientes idosos foram divididos em dois grupos: grupo 1 — parentes de pacientes com demência (n1=48); e grupo 2 — parentes de pessoas sem comprometimento cognitivo (n2=30). Foi-lhes solicitado responder a questionários sobre demência, incluindo o KMAQ.

**Resultados:**

Quando analisadas as pontuações do questionário para os itens de mudanças normais no envelhecimento (g1: 0,53 *vs.* g2: 0,53, valor p: 0,99) não tivemos diferenças significativas no conhecimento entre os dois grupos, não havendo diferenças na pontuação para os itens de mudanças patológicas no envelhecimento (g1: 0,55 *vs.* g2: 0,55, valor p: 0,969).

**Conclusões:**

Estar em contato com a demência não parece melhorar o conhecimento sobre essa síndrome. Conhecer as mudanças normais na cognição durante o envelhecimento permite identificar “sinais de alarme” sugestivos de processos degenerativos, alcançando-se um diagnóstico precoce e mais opções para o tratamento.

## INTRODUCTION

Dementia, also known as *major cognitive disorder*, is a syndrome affecting mainly the elderly, but it is not a “natural” consequence of the physiological aging process. This syndrome causes a decline in cognitive function which is beyond what is expected from normal aging changes, becoming an important cause of disability and dependency among the elderly, which also affects their relatives, caregivers, and society at large, with consequences in all dimensions of a person's life, i.e., physical, psychological, social, and financial^1^.

Drugs and other therapies for dementia developed to date cannot offer a cure but can slow down the neurodegenerative process and/or mitigate the symptoms. However, there is much that can be offered to improve the quality of life of people with dementia and their families, for example, by implementing non-pharmacological interventions based on improving the understanding of this syndrome and its course, as well as providing long-term support^1^.

The World Health Organization (WHO) proposed the “Global Plan of Action in the Public Response to Dementia 2017-2025” to make societies aware of the possibility of working on dementia prevention and on support strategies for caregivers and patients^2^. This plan presents “Support for caregivers” as a strategic action for countries that should develop training and support programs for caregivers and families of patients with dementia, aimed to improve knowledge, skills, and strategies to deal with the patient's symptoms.

Initiatives related to dementia awareness have started in a few countries, where an increase in research and investment in dementia care can be observed^3–7^. With the evidence found in these studies, it is a fact that more actions are required to improve knowledge, awareness, and education about healthy aging and cognitive impairment, in order to reduce modifiable risk factors and reach an earlier diagnosis.

### Questionnaires about dementia knowledge for the population

Some questionnaires have been developed to identify beliefs and perceptions about dementia in relatives and/or caregivers^8–11^. These questionnaires mainly focus on assessing Alzheimer's disease (AD) symptoms and severity. However, an instrument that could also assess normal changes in the aging process might be more useful to determine if people can distinguish between what is a “*normal*” change in the elderly's cognition and what could be a “*red flag.*” The Knowledge of Memory Aging Questionnaire (KMAQ) satisfies this requirement as it aims to assess the knowledge about normal and pathological changes in cognition during the aging process^11^ (Table 1).

**Table 1:** The knowledge of memory aging questionnaire: subdomains of cognition assessed.

Question type: subdomain of cognition		Question number
Normal memory aging items	Memory organization/systems	3–10–20
	Episodic memory phenomena	1–6–17
	Encoding/retrieval factors	2–13–14
	Mnemonics/memory strategies	24–27
	Individual difference/contextual influences	16–21–23
Pathological memory aging items	Types of abnormal deficits	5–11–26
	Identification of abnormal deficits	9–12–19
	Mental health conditions and memory	8–25
	Physical conditions and memory	4–7–28
	Adult dementia/AD	15–18–22

Being developed by Cherry et al. at Louisiana State University, the KMAQ is a *Yes/No/Don't know* questionnaire, containing 28 items, with half of them (14) related to normal cognitive changes in the elderly and the other half (14) asking about non-normative signs in the aging processes that might indicate pathological cognitive impairment (Supplementary material)^11^.

With this questionnaire, it might be possible to assess the general knowledge of people about aging and dementia and also to specify the areas of cognition and aging that are “better” or “worse” known. The KMAQ has been studied in different groups (young, middle-aged, and older adults), and it seems to be well accepted and useful to identify groups that might benefit from an educational program about dementia^12,13^.

The aim of this study was to assess the knowledge about cognition, aging, and dementia by using the KMAQ in people who are in contact with the elderly, with and without cognitive impairment.

## METHODS

This study was approved by the Research and Ethics Committee of the Escola Paulista de Medicina, Federal University of São Paulo (N°: 3.736.135; CEP/UNIFESP N°: 1221/2019). This study was partially funded by the Coordenação de Aperfeiçoamento de Pessoal de Nível Superior – Brasil (CAPES) – finance code 001.

This is a descriptive cross-sectional study, conducted between November 2019 and August 2020, at the Behavioral Neurology Outpatient Clinic and at the Cardiology, Osteomuscular, and Longevity Outpatient Clinic from the Discipline of Geriatrics and Gerontology of São Paulo Hospital, Federal University of São Paulo – Escola Paulista de Medicina (UNIFESP/EPM).

### Participants

Relatives and/or caregivers of elderly patients monitored in these outpatient clinics were classified into the following two groups:

- Group 1: Relatives of patients with dementia or mild cognitive impairment from the Behavioral Neurology Outpatient Clinic (n1=48).
- Group 2: Relatives/caregivers of patients without objective cognitive impairment from the Cardiology, Musculoskeletal, and Longevity Geriatrics Outpatient Clinic (n2=30).

For inclusion, the participants needed to be patients’ relatives, aged 18 years or older, and had attended at least two previous medical consultations. The absence of objective cognitive impairment in group 2 was verified by consulting the patient's medical records of cognitive assessment. All volunteers read and signed an informed consent form before the interview.

### Materials and procedures

We used two kinds of instruments. The first questionnaire was structured by the authors related to demographic information and general questions about cognitive impairment; these results were commented on elsewhere^14^. The second questionnaire was the KMAQ, the object of study in this research. The instructions were clearly explained, and the participants filled out the questionnaires with minimal assistance.

### Statistical analysis

The descriptive results were presented as mean±standard deviation (SD) in order to summarize the demographic characteristics of both groups. One-way ANOVA test was performed to identify statistically significant differences between the means of group 1 and group 2 for each subdomain of items of the scale. If a statistical difference was detected by the ANOVA test, the posterior analysis to identify which group(s) is(are) different was performed with Tukey's HSD/Tukey-Kramer test to specifically assess significant differences between the means of any pair. The normality assumption of the sample was checked based on the Shapiro-Wilk test (α=0.05).

The two-sample t-test was used to identify statistically significant differences between the means of the total scores questionnaire and the adjusted scores questionnaire among groups.

### Scoring process

Results from the KMAQ were expressed as scores, as they were calculated in the original article^11^. There were separate scores for each one of the five subdomains for normal cognitive changes and pathological cognitive changes, so that we could further analyze which areas of cognition were better or worse known. A significance level of 5% was set to reject the null hypothesis, and all statistical tests were two-tailed. Statistical analysis was performed using SPSS version 25.0.

## RESULTS

In a previous publication^14^, we tried to explain knowledge about dementia among relatives of elderly patients in a more qualitative form. The authors structured a questionnaire with items to explore the aspects such as self-initiative to be informed about dementia, the most common causes of cognitive impairment, and sources of information to get documented about dementia. To contextualize this research, we summarized these previous findings:

- Regarding the self-initiative, the results of the Z-test for proportions showed statistical significance (n1:N1=31; proportion=0.65, CI [0.52–0.78] *vs*. n2:N2=10; proportion=0.33 CI [0.24–0.42])p≤0.01); therefore, the relatives of patients with cognitive impairment have more self-initiative to get informed;
- When asked about sources of information, the three most common answers were: doctors and healthcare professionals (n1:N1=39; proportion=0.81 *vs*. n2:N2=26; proportion=0.87); Internet (n1:N1=36; proportion=0.75 *vs*. n2:N2=16; proportion=0.53), and journal/books for both groups (n1:N1=21; proportion=0.44 *vs*. n2:N2=8; proportion=0.27).

In this article, the authors wish to show an objective form of assessing knowledge about dementia by using the KMAQ. In the sections below, the results are presented, analyzing it as a full questionnaire (normal and pathological items) and showing the results of each subdomain of items for each group. A summary of the characteristics of the participants is presented in Table 2. There were no differences between the groups in terms of age, education, or follow-up time.

**Table 2:** Characteristics of relatives/caregivers and summary measures^14^.

		Group 1 (N_1_=48)	Group 2 (N_2_=30)	Sig*
		n_1:_N_1_	n_2:_N_2_	
Gender	Female	37	26	0.27
	Male	11	4	0.27
Profession/occupation related to healthcare		8	3	0.39
Relationship	Child	39	18	0.04†
	Spouse	8	3	0.39
	Siblings	0	2	0.06
	Others	1	7	<0.001†
Caregivers		23	2	<0.001†
		**Mean±SD**	**Mean±SD**	**Sig** ‡
Age		52.31±13.14	55.10±12.06	0.67
Education/schooling		12.73±4.89	13.07±4.40	0.75
Follow-up time		4.54±4.34	6.47±4.35	0.11

Notes: n_1:_N_1:_ number of participants in group 1; n_2_:N_2:_ number of participants in group 2;

* Z-test for proportions results;

† Significant results. SD: standard deviation;

‡ T-test for two independent samples.

### Comparison between the five subdomains of the normal cognitive changes questions of the knowledge of memory aging questionnaire among group 1

One-way ANOVA test was performed to identify significant differences between the means of group 1 among each subdomain of NCQ of the scale (Table 3). The means of all scores in the five subdomains of NCQ did not reach statistically significant differences (p-value>α).

**Table 3:** Comparison between the five subdomains of the normal and pathological questions of the knowledge of memory aging questionnaire among group 1 and group 2.

Subdomains	A Mean±SD	B Mean±SD	C Mean±SD	D Mean±SD	E Mean±SD	F-Statistic	Sig (p-value)
Group 1	0.33±0.21	0.67±0.27	0.67±0.32	0.50±0.27	0.67±0.29	0.30	0.88
Group 2	0.33±0.26	0.67±0.31	0.67±0.29	0.50±0.30	0.67±0.33	0.28	0.89
**Subdomains**	**a Mean±SD**	**b Mean±SD**	**c Mean±SD**	**d Mean±SD**	**e Mean±SD**	**F-Statistic**	**Sig (p-value)**
Group 1	0.67±0.26	0.33±0.25	0.50±0.36	0.67±0.29	0.67±0.22	3.64	<0.00*
Group 2	0.67±0.25	0.33±0.18	0.50±0.29	0.67±0.24	0.67±0.20	2.28	0.06

Notes: A: Memory organization/systems; B: Episodic memory phenomena; C: Encoding/retrieval factors; D: Mnemonics/memory strategies; E: Individual difference/contextual influences. a: Types of abnormal deficits; b: Identification of abnormal deficits; c: Mental health conditions and memory; d: Physical conditions and memory; e: Adult dementia/AD. Here, we see the comparison between the scores of each subdomain of items in both groups. For pathological items in group 1, statistical significance was detected.

* p-value: 0.0062.

### Comparison between the five subdomains of the normal cognitive changes questions of the knowledge of memory aging questionnaire among group 2

One-way ANOVA test was performed (Table 3), showing that the means of all scores in the five subdomains of NCQ were considered equal, without statistically significant differences (p-value>α).

### Comparison between the five subdomains of the pathological cognitive changes questions of the knowledge of memory aging questionnaire among group 1

One-way ANOVA test was performed (Table 3), showing that the means of some scores were not equal, which means the difference between the means of some subdomain scores is large enough to be statistically significant (p-value<α). After analyzing the score means with the Tukey's HSD/Tukey-Kramer test (Table 4), the means of the following pairs are significantly different: x1(a)-x3(c), x3(c)-x5(e). Thus, we might say that relatives and caregivers of the elderly with cognitive impairment (group 1) answered more correctly items related to (a) *Types of abnormal deficits*, (b) *Mental health conditions and memory*, and (c) *Adult dementia/AD*.

**Table 4:** Tukey's HSD/Tukey-Kramer test: comparison between the five subdomains of the pathological cognitive changes questions of the knowledge of memory aging questionnaire among group 1. Subdomains statistically different.

Pair	Difference	SE	Q	Lower CI	Upper CI	Critical mean	p-value
x1-x2	6.73	2.65	2.54	−3.53	16.99	10.26	0.38
x1-x3	10.91	2.65	4.12	0.65	21.17	10.26	**0.031**
x1-x4	1.66	2.65	0.63	−8.60	11.92	10.26	0.99
x1-x5	1.11	2.65	0.42	−9.15	11.38	10.26	1.0
x2-x3	4.18	2.65	1.58	−6.08	14.44	10.26	0.80
x2-x4	5.07	2.65	1.91	−5.19	15.33	10.26	0.66
x2-x5	7.84	2.65	2.96	−2.42	18.10	10.26	0.22
x3-x4	9.25	2.65	3.49	−1.01	19.51	10.26	0.10
x3-x5	12.02	2.65	4.54	1.76	22.28	10.26	**0.012**
x4-x5	2.77	2.65	1.05	−7.49	13.03	10.26	0.95

Notes: Tukey's HSD/Tukey-Kramer test analysis of the scores in Table 3 for pathological items’ score. Some pairs were significantly different: x1(a)-x3(c), x3(c)-x5(e). Interpreting these results, relatives, and caregivers of the elderly with cognitive impairment (group 1) might know more about (a) Types of abnormal deficits, (b) Mental health conditions and memory, and (c) Adult dementia/AD. Bold indicates significant p-values.

### Comparison between the five subdomains of the pathological cognitive changes questions of the knowledge of memory aging questionnaire among group 2

One-way ANOVA test was performed (Table 3), showing that the difference between the means of all score subdomains is not statistically significant, which means the mean scores are equal (p-value>α). Although we recognize that the p-value was very close to the significance level (p-value=0.06), it might be possible that with a larger sample, these results could be significant.

### Comparison between the total and adjusted scores of the normal cognitive changes and pathological cognitive changes questions of the knowledge of memory aging questionnaire among group 1 and group 2

**Figure 1:**
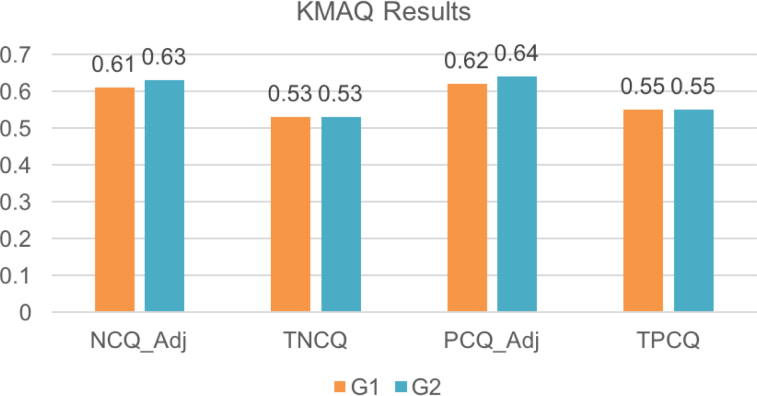
Knowledge of memory aging questionnaire results. We summarize the main results of the scores in both groups. As seen, there were no statistical differences in total scores (considering the full questionnaire), nor in adjusted scores (without “don't know” answers). Being in contact with dementia patients does not seem to improve knowledge among relatives of patients.

The t-test for independent samples was employed to find differences between the groups. These results are available in Table 5 and Figure 1. None of the variables considered for the analyses were significantly different between the groups.

**Table 5:** Descriptive and inferential analysis between group 1 and group 2 for the scores of the knowledge of memory aging questionnaire.

Variables	Descriptive						Inferential				
	Group 1			Group 2			t	SED	95%CID		Sig. (p-value)
	Mean±SD	Min	Max	Mean±SD	Min	Max			Lower	Upper	
NCQ_Adj	0.61±0.15	0.23	0.85	0.63±0.16	0.23	1.00	0.648	0.035	−0.047	0.093	0.519
TNCQ	0.53±0.15	0.21	0.85	0.53±0.17	0.21	0.78	0.007	0.037	−0.073	0.074	0.994
DKA_NCQ	1.96±1.74	0.00	6.00	2.47±2.18	0.00	8.00	1.139	0.446	−0.380	1.397	0.258
PCQ_Adj	0.62±0.14	0.20	0.92	0.64±0.12	0.45	1.00	0.810	0.032	−0.038	0.089	0.421
TPCQ	0.55±0.15	0.14	0.92	0.55±0.12	0.28	0.85	−0.040	0.032	−0.065	0.063	0.969
DKA_PCQ	1.58±1.60	0.00	6.00	2.10±1.92	0.00	7.00	1.286	0.402	−0.283	1.317	0.202

Abbreviations: SD: standard deviation; Min: minimum; Max: maximum; SED: standard error of difference; t: T-statistic or T-value; 95%CID: 95% confidence interval of mean difference; NCQ_Adj: normal memory questionnaire adjusted; TNCQ: total normal memory questionnaire; DKA_NCQ: “don't know” answers in the normal memory questionnaire; PCQ_Adj: pathological memory questionnaire adjusted; TPCQ: total pathological memory questionnaire; DKA_PCQ: “don't know” answers in the pathological memory questionnaire.

The strength of association between variables was verified using the Pearson's correlation coefficient (PCC) and categorized as follows: <0.19=very weak; 0.20–0.39=weak; 0.40–0.59=moderate; 0.60–0.79=strong; and >0.8–1=very strong. The correlation analysis showed some weak (PCC > 0.19 < 0.39) and moderate (PCC > 0.39 < 0.59) correlations between some variables. However, these correlations did not show satisfactory regression values.

The variables considered in this study showed a weak to moderate strength correlation despite the variance and the degree of interaction would not be answered by education or disease duration (independent variables). It might mean that some other non-collected variables would provide a better explanation concerning the dependent-variable variances considered in this research.

## DISCUSSION

It is widely recognized that dementia's prevalence is getting higher. However, the efforts to mitigate its impacts are not growing in the same proportion. According to the WHO^15^, only one-fourth of the countries worldwide have a national policy or strategic plan for supporting people with dementia and their families; the majority of these countries are located in Europe, which contrasts with the recent projections that have shown that dementia's prevalence and incidence will be especially higher in middle- and low-income countries, like those in Latin America or Africa, where the governmental and public health efforts to support these patients are not enough to guarantee early detection, treatment, and quality of life^2^.

Important types of care include primary healthcare, specialist care, community-based services, rehabilitation, long-term care, palliative care, and others, which are also more accessible in high-income countries than in lower income countries, where most dementia care costs are attributable to informal care^5–7^, i.e., care provided by a relative or close friend, generally, without specific training or getting paid to do this job. Furthermore, statistical data revealed that in 2019^1,2^, informal caregivers spent at least 5 h per day providing assistance for daily living activities to a person with dementia at any stage. Due to the financial, social, and psychological stress faced by caregivers, access to information, training services, and social support are highly important.

### Perceptions and beliefs about aging and cognition

A study^16^ surveyed 12 South Asian family caregivers of patients with dementia living in the UK about their previous knowledge of dementia who reported a lack of preparation to recognize early signs of dementia before their relatives got diagnosed, as they assumed that those “memory changes” were related to normal aging.

This lack of preparation might lead to many troubles in relatives’ relationships, especially when behavioral symptoms are predominant and are not linked to a disease. Another study by Godfrey and Townsend^17^ evidenced this problem, as well as the fact that once the person got diagnosed with dementia, his/her relatives felt guilty for blaming the patient for his/her behaviors. This proved the importance of learning about dementia to increase comprehensiveness and reach an early diagnosis so that families might get prepared for the coming manifestations.

An interesting fact evidenced by some studies is the influence of culture on beliefs related to dementia. A study^18^ developed among Muslim communities showed that relatives of patients with dementia considered themselves and their relatives “stigmatized” because their religious community perceived them as being cursed or possessed by evil spirits, causing cognitive and behavioral alterations. Among this community, dementia was seen as a punishment for the family that had a weak faith or had many signs.

Cultural aspects seem to influence more than just beliefs about dementia. Qualitative research evidence suggests that even when family caregivers recognize some symptoms of dementia, they delay seeking medical assessment, going first to ask for help from relatives or friends, maybe because they are afraid of the stigma that a dementia diagnosis causes^19^.

Low and Anstey^20^ assessed the beliefs about causes, risk reduction, and prognosis among 2,000 randomly selected community-dwelling adults who answered a telephone survey. A total of 82% of the interviewees correctly identified “dementia” or “Alzheimer's disease” from a vignette, but there were no differences in terms of recognition rates between the descriptions of mild or moderate dementia symptoms and risk factors. In contrast, other research shows a better comprehension of risk factors for dementia, such as mental disorders, physical and psychosocial stressful situations, diabetes, isolation, and loneliness^19^.

The perceptions about cognitive impairment and dementia may also be linked to social development. In an Irish survey with 1,217 participants, a total of 52% reported that they knew someone with any kind of dementia, although more than 60% were not able to distinguish between early signs of dementia and normal aging cognitive changes^21^. In this study, less than half of the sample (46%) believed that there might be some strategies to prevent dementia by acting on risk factors reduction.

Another proof of the influence of societies on beliefs about cognitive impairment is found in a study among rural communities in South Africa^22^. They reported that elderly women with dementia are considered “witches,” so the community has negative reactions toward them, and these patients and their relatives are banished, stoned, and even killed. This unbelievable thought is also shared by other communities. A qualitative synthesis investigated the attitudes and beliefs among South Asian communities about dementia and found poor knowledge and understanding of dementia, its causes, and its symptoms^23^. Some families thought forgetfulness was part of the normal aging process, and others saw dementia as a punishment from God or a curse caused by demons^23^.

In Brazil, Amado and Brucki^24^ studied the knowledge about AD and its associations with demographic variables such as schooling, profession, and contact with this disease. A total of 1,414 participants answered this survey, with a mean total score for the Alzheimer's Disease Knowledge Scale (ADKS) of 21.6 (SD±3.73), but this score got lower (20.5/30; SD±3.51) when excluding 36.4% of those participants who were healthcare professionals. These results highlighted that educational level and profession influenced positively the score, although being a relative of a patient with AD did not influence the knowledge about the disease.

Another population-based study^25^ evidenced that 20% of family informants/caregivers failed to recognize memory deficits in the elderly with mild dementia, which leads to delays in diagnosis and fewer possibilities for treatment and management plans. To improve dementia literacy, it is necessary to do some observational research to identify perceptions, beliefs, and misconceptions about dementia.

### The knowledge of memory aging questionnaire's utility

Calamia et al.^12^ examined the psychometric properties of the KMAQ. In the study, 933 individuals, ranging in age from 18 to 101 years, answered the questionnaire, and they were college students at Louisiana State University and adults from the community enrolled in the Louisiana Healthy Aging Study (LHAS). For normal aging questions, there were no differences for gender (women: M=6.84, SD=2.13, *vs.* men: M=6.76, SD=2.02; t=0.59, p>.05). In contrast, for pathological aging items, women scored significantly higher (M=8.47, SD=2.45) than men (M=7.89, SD=2.56; t=3.37, p<.05).

In addition, it was observed that scores of knowledge of normal aging decreased with age, but pathological aging was not related to participants’ age. This finding was contrary to the hypothesis of the authors, as it was expected that older individuals would be most familiar with the typical course of memory aging. This evidence suggests that older adults may underestimate signs of cognitive impairment or overestimate normative changes in cognition.

In the present study, the scores of the KMAQ were lower than expected in both groups without statistical differences between them when comparing normal and pathological items scores, despite 12 years of formal education (see “Results”), which is a good level for a Brazilian sample, although we recognized that our sample was much smaller and with a lower level of education than the cited research. Even so, the present findings are in line with previous research exploring dementia knowledge, reflecting the importance of implementing strategies for improving dementia knowledge in the general population, especially among the relatives and caregivers of patients with cognitive impairment.

Other researchers with the KMAQ have shown that healthy older adults have more knowledge about memory aging issues than younger adults, even compared to college students^11^. Also, higher scores at the KMAQ were observed in the healthcare professionals, such as mental healthcare professionals, social workers, caregivers, and senior service providers. The sample of the present study included only a few relatives/caregivers with healthcare occupations, so it was not possible to make this analysis; however, this question should be a key point in future research.

In general, the knowledge about pathological changes is higher than that about normal memory aging issues^11–13^. One reason for this might be that most of the information available in the media is about age-related diseases, such as AD and other dementias, rather than general cognitive health. Similarly, in our study, we observed that relatives of patients with dementia tend to be more familiarized with clinical manifestations of these syndromes (see “Results”). Once again, our findings are in line with the previous research comments, reinforcing the need of thinking about strategies to improve dementia literacy among societies.

This study had some limitations. The follow-up time of the patients involved in the two groups was very variable, as well as the years of schooling of informants. Few participants had a profession/occupation related to the healthcare area. Further studies could assess the knowledge about cognition and aging in these professionals. Larger sample studies could obtain accurate estimates.

In contrast, this study reinforces the need of improving dementia knowledge among the general population. Correcting misinformation could help to develop effective strategies to prevent dementia and mitigate its impacts, such as promotion and education about dementia and its risk and protective factors to motivate healthy changes in lifestyle.

Many pathological signs of dementia are being understood as part of the “normal aging process,” causing delays in diagnosis and reducing the options for treatment. Efforts to improve dementia literacy should be directed toward developing educational and support strategies for families, caregivers, and the general population, so we would be able to recognize what is expected to change in the cognition of the elderly, and what could be a sign of a pathological process such as dementia.

## References

[1] World Health Organization (2021). Dementia [Internet].

[2] World Health Organization (2017). Global action plan on the public health response to dementia 2017–2025 [Internet].

[3] Macdonald M, Martin-Misener R, Weeks L, Helwig M, Moody E, MacLean H (2020). Experiences and perceptions of spousal/partner caregivers providing care for community-dwelling adults with dementia: a qualitative systematic review. JBI Evid Synth.

[4] Zwaanswijk M, Peeters JM, van Beek AP, Meerveld JH, Francke AL (2013). Informal caregivers of people with dementia: problems, needs and support in the initial stage and in subsequent stages of dementia: a questionnaire survey. Open Nurs J.

[5] Forbes DA, Finkelstein S, Blake CM, Gibson M, Morgan DG, Markle-Reid M (2012). Knowledge exchange throughout the dementia care journey by Canadian rural community-based health care practitioners, persons with dementia, and their care partners: an interpretive descriptive study. Rural Remote Health.

[6] Gilhooly KJ, Gilhooly MLM, Sullivan MP, McIntyre A, Wilson L, Harding E (2016). A meta-review of stress, coping and interventions in dementia and dementia caregiving. BMC Geriatr.

[7] Monteiro AMF, Santos RL, Kimura N, Baptista MAT, Dourado MCN (2018). Coping strategies among caregivers of people with Alzheimer disease: a systematic review. Trends Psychiatry Psychother.

[8] Toye C, Lester L, Popescu A, McInerney F, Andrews S, Robinson AL (2014). Dementia knowledge assessment tool version two: development of a tool to inform preparation for care planning and delivery in families and care staff. Dementia (London).

[9] Carpenter BD, Balsis S, Otilingam PG, Hanson PK, Gatz M (2009). The Alzheimer's disease knowledge scale: development and psychometric properties. Gerontologist.

[10] Kuhn D, King SP, Fulton BR (2005). Development of the knowledge about memory loss and care (KAML-C) test. Am J Alzheimers Dis Other Demen.

[11] Cherry KE, Brigman S, Hawley KS, Reese CM (2003). The knowledge of memory aging questionnaire: effects of adding a “don't know” response option. Educ Gerontol.

[12] Calamia M, Reese-Melancon C, Cherry KE, Hawley KS, Jazwinski S (2016). The knowledge of memory aging questionnaire: factor structure and correlates in a lifespan sample. Yale J Biol Med.

[13] Cherry KE, Brigman S, Lyon BA, Blanchard B, Walker EJ, Smitherman EA (2016). Self-reported ageism across the lifespan: role of aging knowledge. Int J Aging Hum Dev.

[14] Montiel-Aponte MC, Bertolucci PHF (2021). Do you look for information about dementia? Knowledge of cognitive impairment in older people among their relatives. Dement Neuropsychol.

[15] Wordl Health Organization (2021). World failing to address dementia challenge [Internet].

[16] Adamson J (2001). Awareness and understanding of dementia in African/Caribbean and South Asian families. Health Soc Care Community.

[17] Godfrey M, Townsend J (2001). Caring for an elder with dementia: The experience of Asian caregivers and barriers to the take up of support services.

[18] Mackenzie J (2006). Stigma and dementia: East European and South Asian family carers negotiating stigma in the UK. Dementia.

[19] Mukadam N, Cooper C, Basit B, Livingston G (2011). Why do ethnic elders present later to UK dementia services? A qualitative study. Int Psychogeriatr.

[20] Low F, Anstey KJ (2009). Dementia literacy: recognition and beliefs on dementia of the Australian public. Alzheimers Dement.

[21] Glynn RW, Shelley E, Lawlor BA (2017). Public knowledge and understanding of dementia-evidence from a national survey in Ireland. Age Aging.

[22] Mkhonto F, Hanssen I (2018). When people with dementia are perceived as witches. Consequences for patients and nurse education in South Africa. J Clin Nurs.

[23] Hossain M, Crossland J, Stores R, Dewey A, Hakak Y (2020). Awareness and understanding of dementia in South Asians: a synthesis of qualitative evidence. Dementia (London).

[24] Amado DK, Brucki SMD (2018). Knowledge about Alzheimer's disease in the Brazilian population. Arq Neuropsiquiatr.

[25] Lam K, Chan WSY, Luk KHJ, Leung AYM (2019). Assessment and diagnosis of dementia: A review for primary healthcare professionals. Hong Kong Med J.

